# The Coda of the Transient Response in a Sensitive Cochlea: A Computational Modeling Study

**DOI:** 10.1371/journal.pcbi.1005015

**Published:** 2016-07-05

**Authors:** Yizeng Li, Karl Grosh

**Affiliations:** 1 Department of Mechanical Engineering, University of Michigan, Ann Arbor, Michigan, United States of America; 2 Department of Biomedical Engineering, University of Michigan, Ann Arbor, Michigan, United States of America; University of California at Berkeley, UNITED STATES

## Abstract

In a sensitive cochlea, the basilar membrane response to transient excitation of any kind–normal acoustic or artificial intracochlear excitation–consists of not only a primary impulse but also a coda of delayed secondary responses with varying amplitudes but similar spectral content around the characteristic frequency of the measurement location. The coda, sometimes referred to as echoes or ringing, has been described as a form of local, short term memory which may influence the ability of the auditory system to detect gaps in an acoustic stimulus such as speech. Depending on the individual cochlea, the temporal gap between the primary impulse and the following coda ranges from once to thrice the group delay of the primary impulse (the group delay of the primary impulse is on the order of a few hundred microseconds). The coda is physiologically vulnerable, disappearing when the cochlea is compromised even slightly. The multicomponent sensitive response is not yet completely understood. We use a physiologically-based, mathematical model to investigate (i) the generation of the primary impulse response and the dependence of the group delay on the various stimulation methods, (ii) the effect of spatial perturbations in the properties of mechanically sensitive ion channels on the generation and separation of delayed secondary responses. The model suggests that the presence of the secondary responses depends on the wavenumber content of a perturbation and the activity level of the cochlea. In addition, the model shows that the varying temporal gaps between adjacent coda seen in experiments depend on the individual profiles of perturbations. Implications for non-invasive cochlear diagnosis are also discussed.

## Author Summary

The fluid-structure-electrical interaction in the cochlea enable the basilar membrane, one of the most important structures in the cochlear partition, to display different dynamic patterns depending on the frequency content of the incoming sound. Interestingly, in a healthy cochlea the motion of the basilar membrane shows echoes upon an impulse acoustic stimulation delivered to the ear canal. The delay, duration, and shape of these echoes vary from one cochlea to another. A hypothesis that irregularities of the properties of the cochlear partition coherently scatter acoustic waves and generate echoes is examined. These irregularities are posited to arise, for example, the damage of the sensory cells or the natural randomness in the morphology of the cochlear partition. Here we build a physiologically-based mathematical model to understand the echoes observed in experiments by introducing irregularity to the properties of the sensory cells. We found that the patterns of the echoes depend on the individual profiles of the irregularities. Our work suggest that the ear canal recording, which is correlated to the dynamics of the basilar membrane, can be used as a non-invasive tool not only to diagnose the intracochlear damage but also to interpret these data given its idiosyncratic origin.

## Introduction

**Fig 1:**
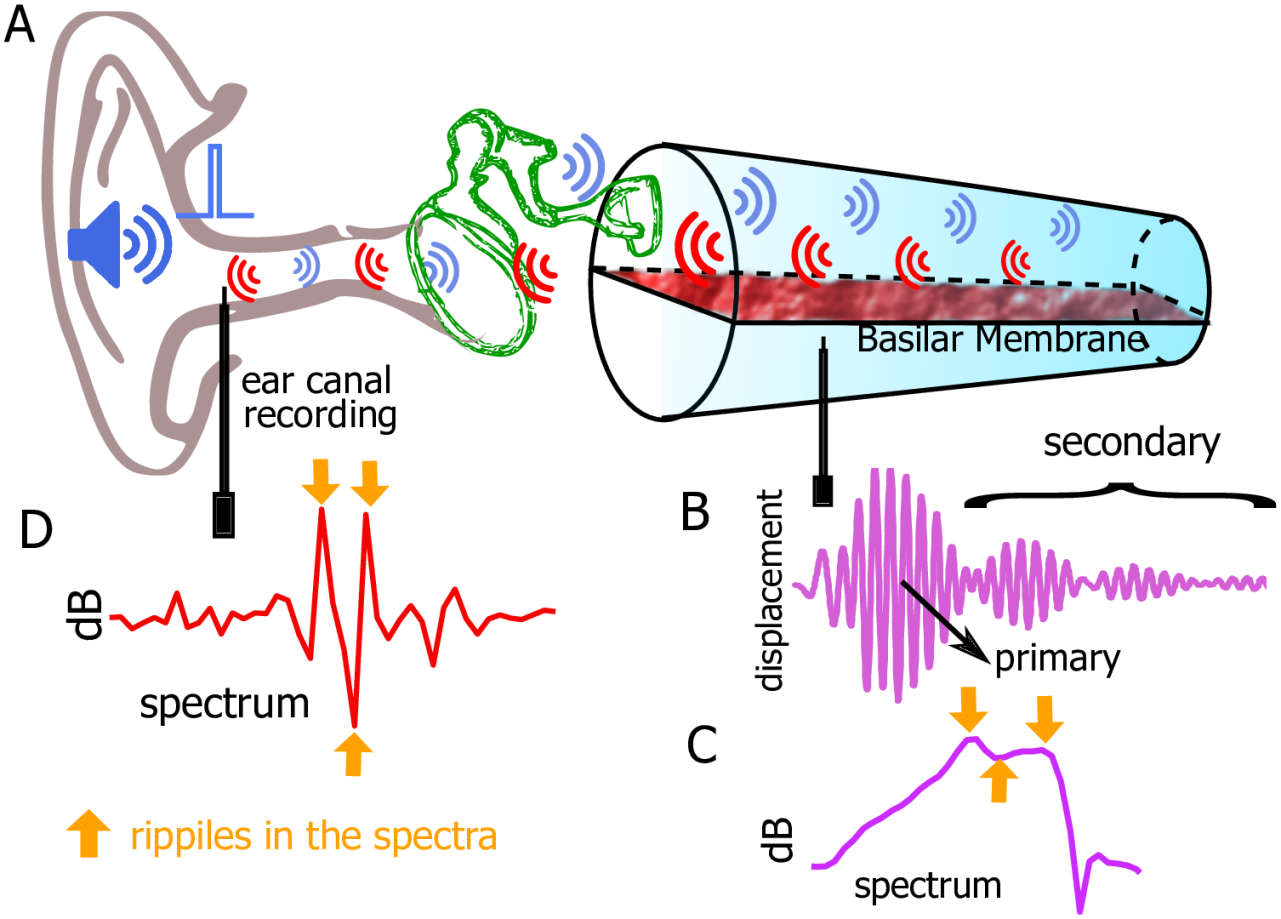
Schematics of two signal pathways in the peripheral auditory system and sample responses. A: Schematics of two signal pathways. One is the conventional acoustic path where sound pressure is transmitted to the cochlea via the tympanic membrane and the middle ear bones. The other is the otoacoustic emission pathway where sound is generated inside the cochlea and can be measured in the outer ear canal with a probe. B: Upon an impulse acoustic simulation, the basilar membrane (BM) displays not only a primary impulse response but also a coda of delayed secondary responses. The coda typically lasts for 3–5 ms depending on the longitudinal location of the BM and the health of the cochlea. C: The corresponding spectrum of the BM to the temporal response in B. The frequency may vary among all the audible frequency ranges depending on the longitudinal location of the BM. The spectrum is characterized by ripples. D: A sample spectrum of the ear canal recording shows ripples. The ripples in C and D would disappear if the BM does not exhibit a coda. The data in B, C, and D are from Shera and Cooper [3].

In a sensitive cochlea, the response of the basilar membrane (BM) to transient external acoustic excitation consists not only of a primary impulse response but also of a coda of delayed secondary responses (sometimes called echoes or ringing) with varying amplitudes but similar spectral content centered near the best frequency of the measurement location [1–3] (see Fig 1A and 1B for an illustration). The coda is physiologically vulnerable, disappearing when the cochlea is compromised. Ripples in the spectra of the BM motion and in the ear canal recording of otoacoustic emissions (OAEs) [4, 5] (see Fig 1C and 1D) are the frequency domain correlate of the repetitious wave packets of the coda in the time domain. The temporal coda or spectral ripples can be evoked by transient acoustic stimuli, acoustic tone bursts, or electrical stimuli [4, 6–9].

Depending on the individual cochlea and location of the measurement, the temporal gap between the primary impulse and coda ranges from once to thrice the group delay of the primary impulse (the group delay of the primary impulse is on the order of a few hundred microseconds) [1–3]. For example, in click-evoked BM responses from the base of the chinchilla cochlea [1], the temporal gaps were less than twice the primary group delay. They also observed secondary coda with long or extended tails when the sound pressure levels were low. Similar temporal gaps between adjacent wave packets were found by Shera and Cooper [3]; in addition, the inter-packet delays were not always constant, sometimes displaying a wax and wane. This intricate behavior is not completely understood.

The echoes of the coda will have an impact on evoked OAEs either in the time or in the frequency domain. Because OAEs are nearly universally used in newborn hearing screening and in many noninvasive hearing exams [10], it is important to understand OAE generation and link measurable features to a physiological cause. However, the complete correlation of the spectral or temporal details to their intracochlear sources is still lacking. Kemp, who provided the first observation of the secondary temporal response to a click in the ear canal, suggested that the emissions arise from physical properties of the inner ear such as the outer hair cells (OHCs) [4]. Electromotility in the cell body of the OHC due to the protein prestin was required for normal low level evoked OAEs [11], although high level OAEs are still evoked in prestin-null animals with operating mechano-electro transduction (MET) channels. Hence an intracochlear source is needed to generate the OAE. Irregularity in the properties of the cochlea (sometimes called roughness) has been proposed as a potential contributor to the spacing of peaks in the OAE spectra as early as 1983 [12]. The relation between the OAE spectra and the irregularities of the cochlear partition was theoretically analyzed [13] and lead to the development of the coherent reflection theory. In this theory, intracochlear waves scattered by random irregularities are shown to give rise to constructive or destructive interference that contributes to ripples in OAE spectrum [3, 8, 14]. This theory has been studied extensively in the context of a one dimensional model. Although the existence or source of these random perturbations remains to be conclusively identified [3], they have been variously attributed to small variations in MET channels or to OHC electromotility (e.g. [15]). Meaud and Lemon [15] showed that random perturbations in OHC electromechanical coupling can give rise to a coda under normal acoustic excitation using a mathematical model, but the different realizations of the randomness or the cochlear response to internal excitation were not investigated. Using experimental results, Shera and Cooper [3] found a strong correlation between ripples in the BM response spectrum and those in the OAE spectrum indicating that these two phenomena are not independent. Factors that affect the BM response also affect the fluid mechanical input to the inner hair cell stereocilia, the ultimate mechanical excitation before neural stimulation occurs. It has been posited that the gaps in the coda may also be used by the auditory periphery to provide input on gaps in temporal encoding [9]. Further, it has been hypothesized that roughness and the resultant spectral rippling may also actually determine our ability to discriminate between different frequencies [16]; a hypothesis that could help explain individual differences in such tasks.

Under normal circumstances, the cochlea is stimulated by external acoustic excitation, which generates a disturbance propagating on the BM from the base to the apex. OAEs are controlled by force generation inside the cochlea (either through nonlinearities or OHC mediated active force generation). However, less is known when the cochlea is internally excited. In this work, we used a physiologically-based, mathematical model to study the cochlear response with either random or focal perturbations under internal and external excitation. The acoustic waves and the associated traveling and delay times will be analyzed in detail. In particular, we investigated the effect of different random perturbations to understand their role on the diverse secondary cochlear response. With different profiles of perturbation, our model predicted diverse temporal delays between adjacent coda seen in experimental measurements. Implications on non-invasive cochlear diagnosis are discussed as well.

## Mathematical and Computational Model

We used a finite element approximation of a physiologically-based, mechano-electro-acoustical model modified from [17, 18] as in [19] for a gerbil cochlea to simulate the response of the cochlea to internal and normal external stimulation. The model includes the following components: compressible fluid dynamics, kinematics and kinetics of the organ of Corti (OoC), dynamics of the tectorial membrane (TM) and the BM, a longitudinally coupled viscoelastic model of the TM, longitudinal coupling of the electric conduction in the scala vestibuli (SV) and scala tympani (ST), electromotility of the outer hair cells, and conductance of the stereocilia. The model supports both compressional waves and fluid-structure coupled traveling waves whose directions are not *a priori* chosen, but rather dictated by the underlying physics. A list of selected parameters are given in Table 1. The rest of parameters can be found in S1 Table as well as in [17–19].

**Table 1:** Selected parameters for the cochlear model (*x* is in meters measured from the base of the cochlea). BM: basilar membrane. HB: hair bundle. MET: Mechanoelectrical transducer. OW: Oval window. RW: Round window.

Parameters	values
*β* (40°C)	385 × 10^−6^ /K
*c*_*p*_ (40°C)	4.178 J/gK
*α*	0.3
${\dot{Q}}_{0}$	0.5 W
*t*_0_	30 *μ*s
Duct height	0.5 mm
BM length	11 mm
OW stiffness	1.8 × 10^8^ N/m per unit area
OW damping	5.8 × 10^2^ N ⋅ s/m per unit area
RW stiffness	1.8 × 10^7^ N/m per unit area
RW damping	5.8 × 10^2^ N ⋅ s/m per unit area
MET sensitivity ($G_{a}^{1}$)	(3.6016 S/rad) (*L*_HB_|_*x* = 0_/*L*_HB_)*e*^−(252.3/m)*x*^ per unit length
HB length (*L*_HB_)	1 *μ*m (base) to 6 *μ*m (apex)

**Fig 2:**
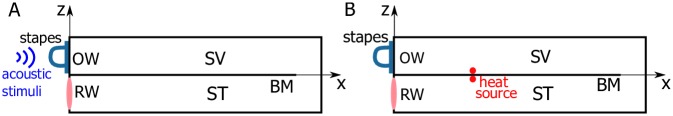
Schematics of the two excitation methods and the coordinate system used in the model. A: Impulse acoustic stimulation delivered at the stapes. B: Transient intracochlear heating from laser light absorption. The two points above and below the BM represent two effective absorption locations. OW: Oval window. RW: Round window. BM: basilar membrane. SV: scala vestibuli. ST: scala tympani.

We used two excitation methods to study the effect of perturbations on the cochlear response. The first, shown in Fig 2A, was an impulse acoustic stimulation delivered at the stapes which in turn excites the intracochlear fluids (e.g., [20]). This method represents the conventional acoustic pathway where sound travels from the ear canal into the cochlea. The second stimulation paradigm, shown in Fig 2B, was transient intracochlear heating from laser light absorption. This approach of cochlear excitation was studied experimentally and theoretically in [19]. Transient heat deposition was modeled as an inhomogeneous forcing in the wave equation as in [19, 21]:

$$\nabla^{2}p-\frac{1}{c^{2}}\frac{\partial^{2}p}{\partial t^{2}}=-\frac{\beta }{c_{p}}\frac{\partial }{\partial t}\dot{q}\;,$$ (1)

where *c* is the speed of sound in the fluid, *β* is the coefficient of volume thermal expansion of the fluid, *c*_*p*_ is the specific heat capacity of the fluid at constant pressure, and $\dot{q}$ is the heat absorbed by the fluid per unit time and per unit volume. Only when the rate of heat addition changes is there an acoustic excitation. Upon simplification, the transient laser source [19], $\dot{q}$, can be represented as a boxcar function $\dot{q}=\alpha {\dot{Q}}_{0}\delta (x_{0})\left[H(0)-H(t_{0})\right],$ where ${\dot{Q}}_{0}$ is the power of the laser, *α* is the estimated fraction of power absorbed by the fluid [19], **x**_**0**_ is the position of the laser focal point, *H*(*t*) is the Heaviside function in time, and *t*_0_ is the duration of the transient laser power. When a laser was focused on the BM, the surrounding fluid above and below the BM is heated as well. In this case, we consider two effective heat absorption points as illustrated in Fig 2B, and the right hand side of Eq 1 will have two $\dot{q}$ terms. The strength of the heat absorption at the two points can be either equal (symmetric) or non-equal (asymmetric), depending on the exact laser focal position. Under typical laser light excitation, the heat absorption was slightly asymmetric (above and below the BM), as discussed in [19].

In order to perturb the smooth model, we introduced random but very small fluctuations in the sensitivity of the mechanoelectrical transducer (MET) channel. The MET sensitivity is the slope of the change of the conductance of the hair bundle (HB) with respect to the HB’s rotation (*θ*_*hb*_) [17, 20]. Along with the somatic electromotility, this parameter is a main determinant of the electromechanical feedback responsible for enhancement of the cochlear response to low level sounds. At these levels, we assumed that the conductance of the HB is a linear function in *θ*_*hb*_ (more discussion can be found in [18]). $G_{a}^{1}\left(x\right)$ represents the unperturbed, smooth activity level of the cochlea, where *x* is the longitudinal distance away from the stapes [see $G_{a}^{1}\left(x\right)$ in Table 1]. In a perturbed system, we prescribe additional variations on $G_{a}^{1}\left(x\right)$. The level of full, 100% activity (or the MET sensitivity) was determined from experiments so that the predicted BM gain matches experimental data while still remains stable [19, 20]. When perturbations exits, they perturb the maximum level of MET channel sensitivity and lead to an unstable model. Hence, an activity level of 90% was used in all simulations to avoid perturbation–induced instability.

**Fig 3:**
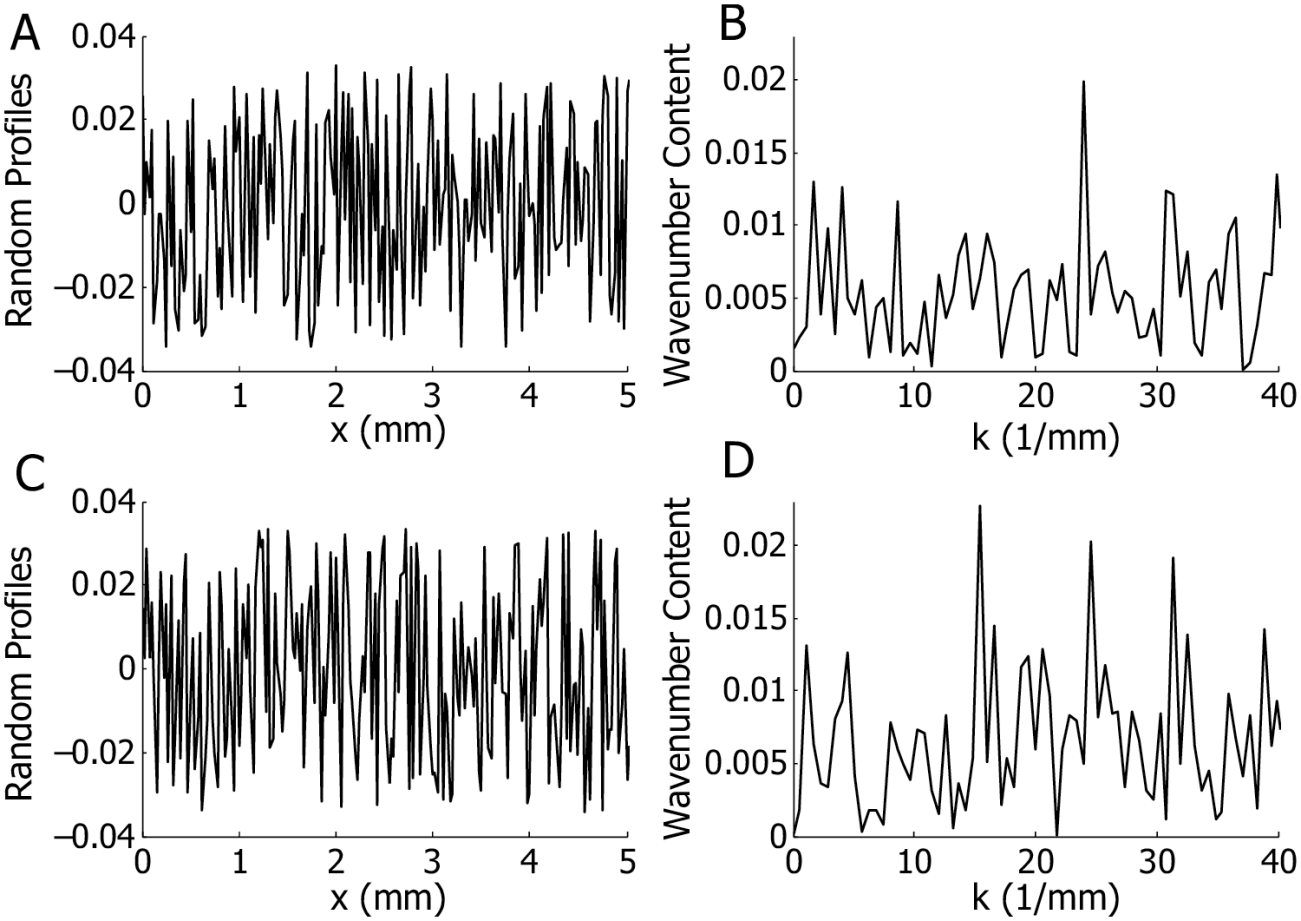
Random profiles for in the spatial and in the wavenumber domains. A and B: Random profile for R1. C and D: Random profile for R2. A and C: Random profiles in the spatial domain. The profiles within the initial 5 mm from the stapes are illustrated. The amplitudes are shown for random variations with standard deviations 0.02. B and D: Random profiles in the wavenumber domain. The profile is shown for wavenumber less than 40 mm^−1^, which corresponds to a wavelength greater than *λ* = 2*π*/*k* = 157 *μ*m.

We use uniformly distributed random perturbations (with average 0) generated by the rand command in MATLAB^©^. A 25 *μ*m mesh is used for the finite element model, which also determines the spatial resolution of the random profiles; i.e., the random numbers added to $G_{a}^{1}\left(x\right)$ is right on each finite element node. In this model, we use 2% level of random perturbation, which means the standard deviation of the collective random numbers is 0.02. Two random profiles (denoted as R1 and R2) were chosen as exemplars as they are able to produce representative cochlear responses among the five random profiles we have tested. Fig 3A and 3C shows the random profiles of R1 and R2, respectively, in the spatial domain for the first 5 mm from the cochlear base. The random profile can also be presented in the wavenumber (*k*) domain, which is obtained by the Fourier transformation of the random profile in the spatial domain and multiplied by 2*π* (by the definition of wavenumber). Fig 3B and 3D shows the content of R1 and R2, respectively, in the wavenumber domain for *k* = 2*π*/*λ* < 40 mm^−1^. In addition to the random profiles described above, a focal MET perturbation was used to represent morphological variations such as OHC missing at a given location. In this case, the perturbation was a Dirac delta function in space.

## Results

### Distributed and focal perturbations both give rise to a coda

**Fig 4:**
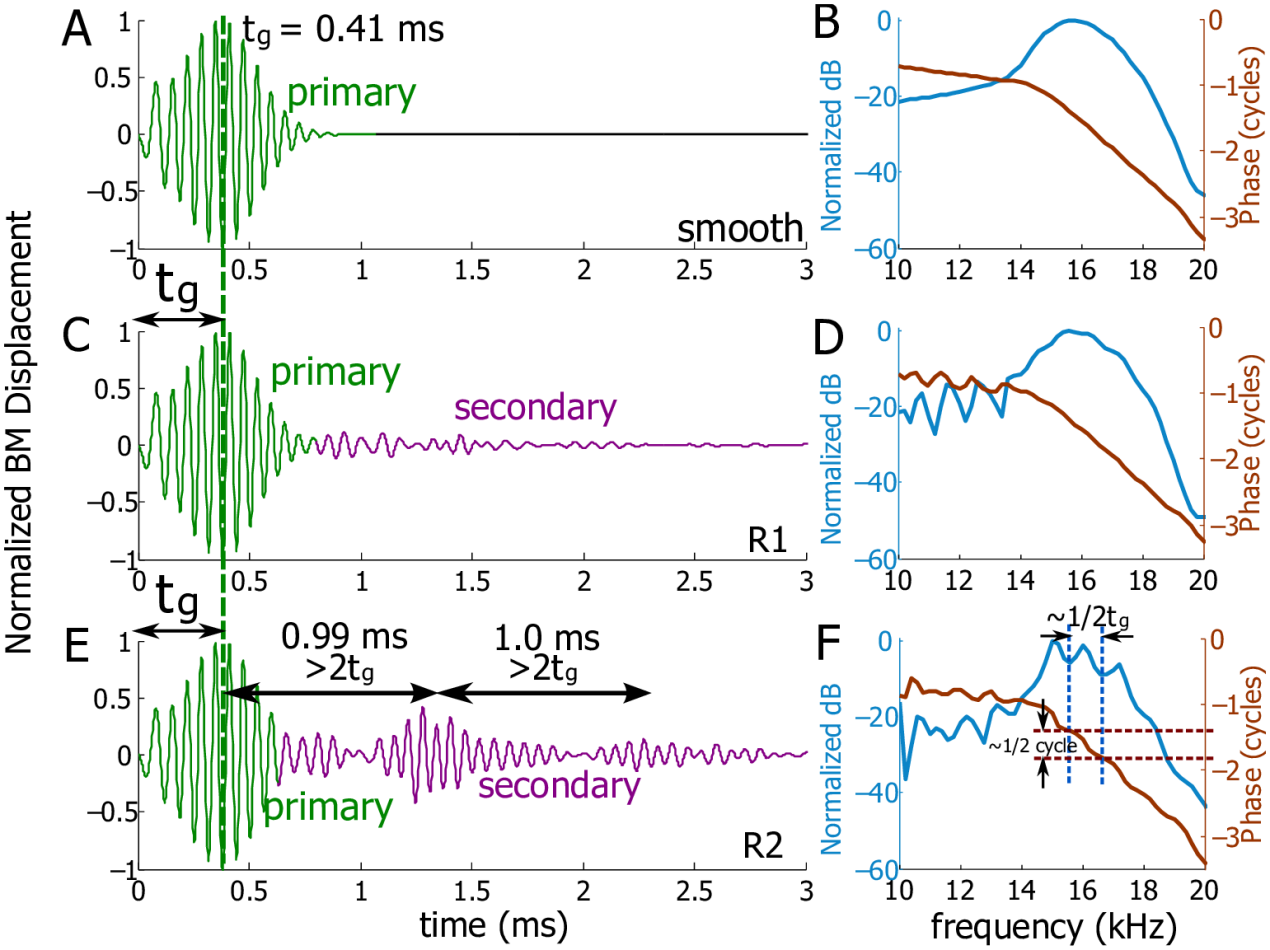
Smooth and perturbed BM responses at 2.5 mm under acoustic stimuli. A: Normalized temporal displacement of the BM for a smooth cochlea. *t*_*g*_ is the group delay of the primary wave packet. B: Spectrum of the BM from A. The amplitude is normalized to its maximum value. C: Normalized temporal displacement of the BM for a cochlea with random perturbation R1 added to the MET channel. D: Spectrum of the BM from C. E: Normalized temporal displacement of the BM for a cochlea with random perturbation R2 added to the MET channel. The displacement normalization ratio of C to E is 0.89. F: Spectrum of the BM from E. The vertical dashed lines enclose a ripple in the BM amplitude around the best frequency. The horizontal dashed lines show corresponding phase jump (approximately half cycle) for the ripple in the amplitude.

For a smooth, unperturbed cochlea, the normalized temporal BM displacement at 2.5 mm under an impulse acoustic simulation (shown schematically in Fig 2A) is shown in Fig 4A. The primary wave packet is consistent with experimental results, evincing a variable amplitude and frequency content, the latter of which is known as the glide as the peak frequency increases with time over the duration of the wave packet. The group delay of this wave packet (denoted as *t*_*g*_) was obtained either from the slope of the phase in the spectrum (Fig 4B) or the ‘center of gravity’ of the wave packet in the time domain, indicated by the vertical dotted line in Fig 4A. Here the group delay was found to be 0.41 ms. *t*_*g*_ varies with longitudinal location and depends on the health of a cochlea. The spectrum shows the best frequency of the BM at 2.5 mm, ∼16 kHz (Fig 4B). The smooth spectrum and a single wave packet are hallmarks of an unperturbed cochlear model at all spatial locations for our linearized model.

Under R1 or R2, our model predicts secondary responses (the coda) after the primary one under external acoustic stimulation (Fig 4C and 4E). When random perturbation R1 (Fig 3A) is prescribed, the secondary response is predicted to be small and does not form regular wave packets (Fig 4C). The distributed perturbation also produces ripples in the spectrum at frequencies below the best frequency (Fig 4D). When random perturbation R2 (Fig 3C) is prescribed, the secondary response exhibits clear wave packets, the frequency content of which is centered at the best frequency of this location on the BM. The group delay of each secondary wave packet can be obtained from the phase in the spectrum of just that wave packet (with others removed from the time domain response). From the group delays we get the temporal gaps between adjacent wave packets. In Fig 4E, the two temporal gaps are both ∼ 1.0 ms, a value greater than 2*t*_*g*_ = 0.82 ms. The spectrum ripples produced by R2 are more remarkable around the best frequency, and each ripple corresponds to a phase jump close to half a cycle (see the dashed lines in the Fig 4F). These results show that the duration of the coda is usually a few milliseconds and its organization and intensity depend on the nature of the roughness. Even in the presence of random perturbations, the group delay of the primary wave packet remains preserved at 0.41 ms (see Fig 3A, 3C and 3E).

**Fig 5:**
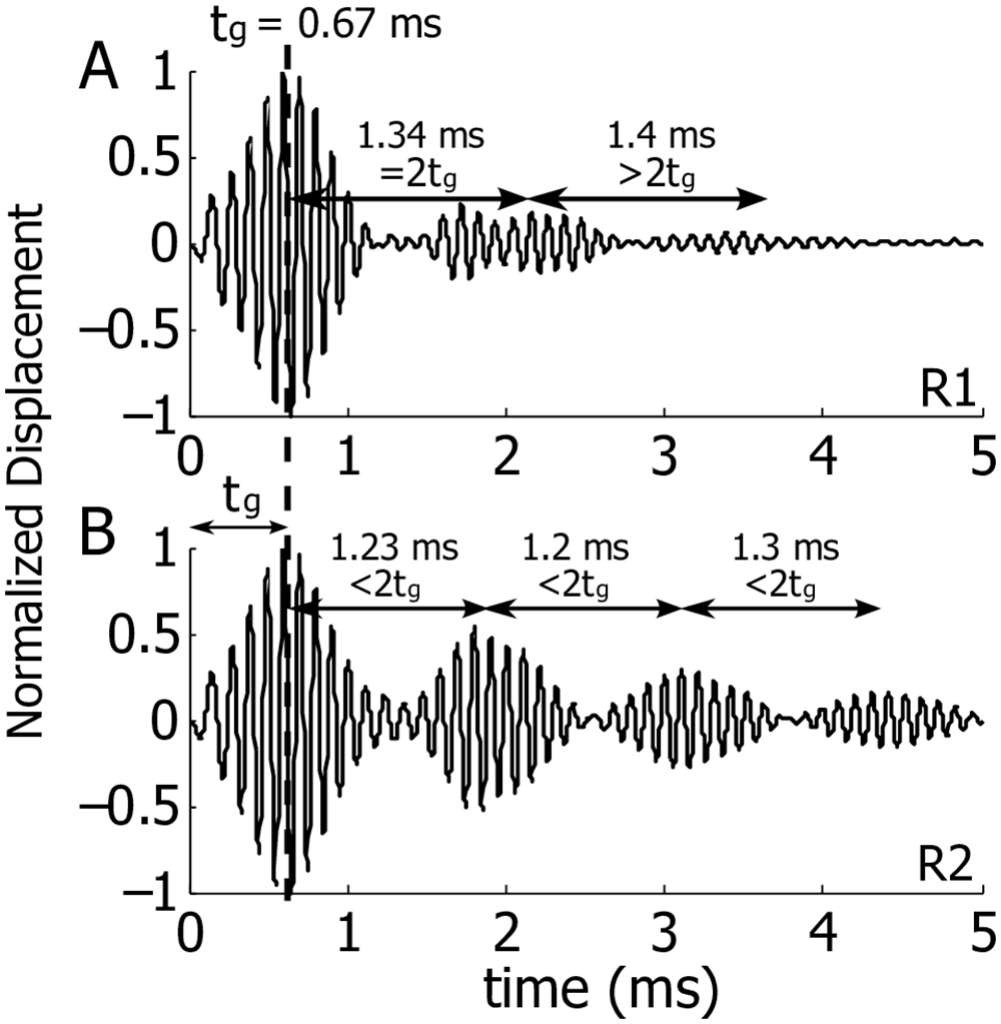
Normalized spatial BM displacements under acoustic stimuli with distributed random perturbations on the MET channel. BM responses at 4.0 mm, where the group delay is 0.67 ms. A: Response with random profile R1. B: Response with random profile R2. The displacement normalization ratio of A to B is 1.09.

The effects of the same random profiles can be very different at different longitudinal locations. Fig 5 shows the predicted response of the BM at 4.0 mm, where the group delay is 0.67 ms. Random profile R1 generates small, ill-defined wave packets at 2.1 ms and 3.5 ms (Fig 5A). The temporal gap between the primary and first coda wave packet is 2*t*_*g*_ = 1.34 ms and the following gap is 1.4 ms, more than 2*t*_*g*_. The echoes produced by random profile R2 have a more systematic pattern with group delays 1.9 ms, 3.1 ms, and 4.4 ms (Fig 5B). In this case, the temporal gaps are 1.23 ms, 1.2 ms, and 1.3 ms, all being less than 2*t*_*g*_.

**Fig 6:**
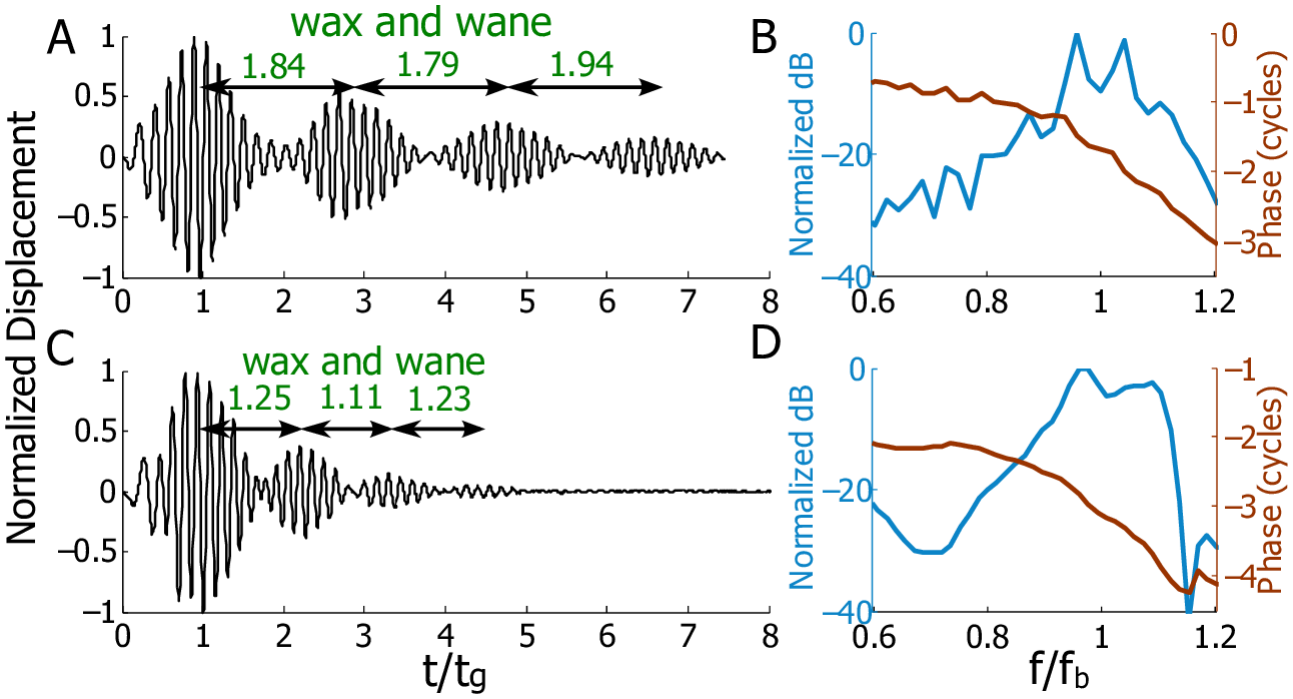
Comparison of experimental results with theoretical predictions for BM displacement. A-B: Model prediction. C-D: Experimental results from Fig 9 of Shera and Cooper [3]. The original data were obtained from the authors. A and C: Temporal displacements of the BM plotted versus non-dimensionalized time with respect to each group delay. A is a rescaled version of Fig 5B. Normalization factors: 0.67 ms for the theoretical prediction and 0.91 ms for the experimental data. B and D: BM spectra. The frequency scale is normalized with respect to each individual best frequency. Normalization factors: 9.6 kHz for the theoretical prediction and 7.2 kHz for the experimental data.

In Fig 6 we compare our predictions (acoustic stimulation with 2.0% R2, Fig 6A and 6B) to experimental results from Shera and Cooper (Fig 6C and 6D) [3]. To enable a comparison between the two results (model versus experiment, different species, BM locations, and random profiles), we normalize the time to the group delay and the frequency to the best frequency of the measurement location for both the experimental and theoretical results. In both the experimental result and the theoretical prediction, temporal gaps between each pair of adjacent wave packets undergo wax and wane (Fig 6A and 6C): from 1.84 decreased to 1.79 and then increased to 1.94 in the simulation; from 1.25 decreased to 1.11 and then increased to 1.13 in the experiment. In the frequency domain (Fig 6B and 6D), two peaks are present around the best frequency in each spectrum; these ripples characterize the BM response in a perturbed cochlea with temporal coda. While the predicted and experimental results differ quantitatively, the qualitative similarities are striking as both responses display a waxing and waning of the secondary responses.

**Fig 7:**
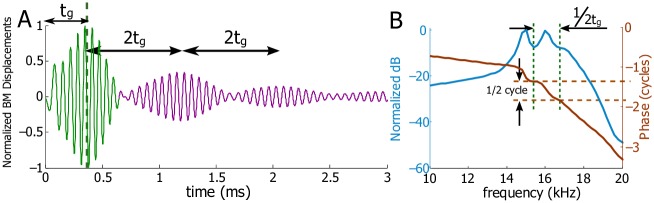
A focal perturbation of the MET channels gives rise to an well-formed and periodic coda. A: Normalized temporal displacement of the BM at 2.5 mm for a cochlea with 12% focal activity increment on the MET channel at 2.5 mm. B: Spectrum of the BM from E. The phase jump for a ripple is exactly half cycle. The echoes and ripples fade with the decreasing activity level of the cochlea or with the increasing longitudinal distance (about 0.75 mm) away from the focal perturbation.

If a single, focal, and more intense fluctuation (a point 12% variation of the MET channel at 2.5 mm) is used, our model predicts that the secondary responses show a remarkable temporal pattern when the BM is measured at the same longitudinal location as the focal fluctuation. All the adjacent echoes have similar waveforms as the primary one and are separated precisely by 2*t*_*g*_ (Fig 7A), i.e., the group delays of the echoes are *t*_*g*_, 3*t*_*g*_, 5*t*_*g*_, 7*t*_*g*_, ⋯. In the spectrum, ripples only appear around the best frequency (Fig 7B) and can be quantified in two ways. The frequency gap between adjacent ripples is related to the temporal gap of adjacent echoes, i.e., 1/2*t*_*g*_ ≃ 1.25 kHz; the phase jump associated with each ripple is half a cycle. The echoes and ripples fade with the decreasing activity level of the cochlea or with the increasing longitudinal distance (about 0.75 mm) away from the focal perturbation. This intentional focal fluctuation (where a stainless steel bead was used to load the BM) was discussed in [3], but no temporal results were presented.

### The coda is preserved under asymmetric intracochlear heating

**Fig 8:**
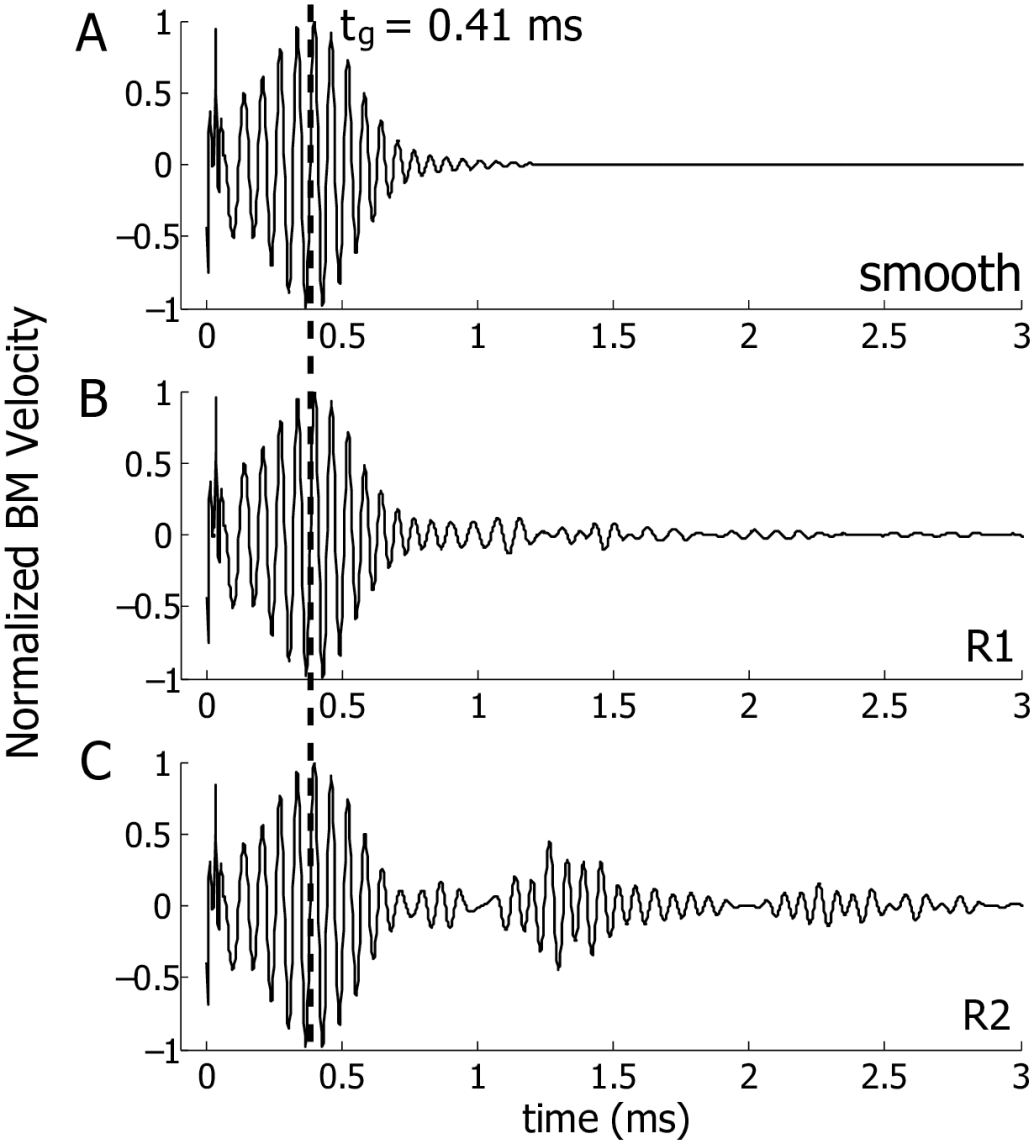
Normalized velocities of the BM at 2.5 mm under a pair of slightly asymmetric heating source on the BM at 2.5 mm. The duration of the heating is 30 *μ*s. The heating strength ratio below and above the BM is 0.52:0.48. A: Using a smooth cochlea model. B: Adding the R1 perturbation profile at 2.0% to the MET channel. C: Adding the R2 perturbation profile at 2.0% to the MET channel.

Now we consider the BM response under intracochlear heating (Fig 2B). When the heating strength is asymmetric across the cochlear partition (probably the case in the experiments [19]), a resultant force is generated on the BM [19], giving rise to an initial spike of the BM, which decays quickly after the heat is removed (Fig 8). More details on the generation of this spike can be found in an earlier work [19].

Here we present results for a pair of asymmetric, focal, and transient heating sources at 2.5 mm with a heat absorption ratio below and above the BM of 0.52:0.48 (a value which gave an initial spike seen similar to that seen in experiments [19]). For a smooth cochlea, the asymmetric heating generates only a primary wave packet with the identical group delay, *t*_*g*_, and similar waveform as the one from an acoustic simulation (compare Figs 8A and 4A). When random profiles R1 and R2 are added (Fig 8B and 8C), the features of the secondary response are very similar to those seen under acoustic excitation (Fig 4C and 4E). The lack of the well-structured coda in one case (Fig 8B) and the presence of it in another (Fig 8C) is in accordance with the prediction from acoustic simulation. If the BM is measured away from the focal laser excitation, the initial sharp peak at *t* = 0 will disappear but the coda is still present in a sensitive preparation (compare to our simulations due to acoustic simulation presented in Figs 4 and 5).

### The response of the stapes reflects the internal perturbation

**Fig 9:**
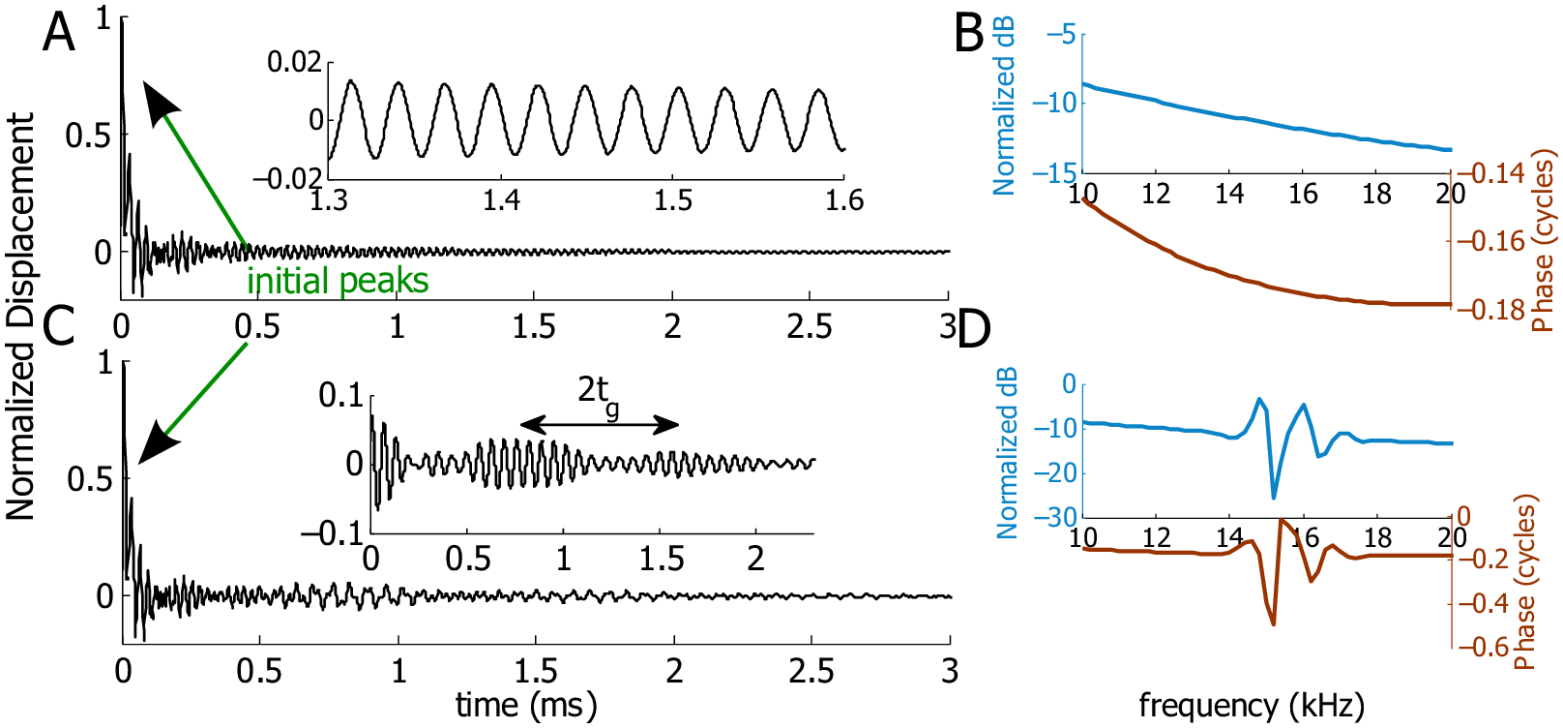
Response of the stapes under an impulse acoustic stimulation for a smooth and a focally perturbed cochlea. A-B: Model prediction from a smooth cochlea. A: Normalized temporal displacement of the stapes. The inset is the zoomed-in response showing the small oscillations of the stapes as an effective damped oscillator. B: Spectrum of the stapes (the amplitude has been normalized to its maximum value). C-D: Model prediction from a focally perturbed cochlea where 12% activity increment is added to the MET channel at 2.5 mm. C: Normalized temporal displacement of the stapes. The inset shows the filtered temporal response of the stapes: the spectrum content between 14 kHz and 18 kHz is retained and the others are filtered. D: Spectrum of the stapes (the amplitude has been normalized to its maximum value). Ripples are present around 16 kHz, the best frequency of the perturbed location.

The stapes also displays an extended response related to the coda. Since the stapes is part of the middle ear, this response would subsequently evoke an OAE. Under an impulse acoustic stimulation, the stapes is forced at *t* = 0 and then undergoes free motion determined by the properties of the cochlea. The forcing gives rise to an initial peak at *t* = 0 that decays quickly within about 0.1 ms (Fig 9A and 9C). For a smooth cochlea, the response of the stapes after the initial peak operates effectively as a damped oscillator: the oscillations have decreasing amplitudes in time and a fixed frequency (see the inset of Fig 9A). The spectrum is smooth everywhere and only has gradual decrease in amplitude (Fig 9B).

For a cochlea focally (or locally) perturbed at 2.5 mm, the stapes exhibits secondary responses as well (Fig 9C). Ripples in the spectrum are prominent around 16 kHz, the best frequency at 2.5 mm where the MET channel activity is perturbed (Fig 9D). We band filtered the spectrum with a square window from 14 kHz to 18 kHz and obtained clear wave packets in the temporal domain, as shown in the inset in Fig 9C. Remarkably, the temporal gap between these wave packets is also twice the group delay, 2*t*_*g*_, of the primary wave packet at 2.5 mm under acoustic stimuli (see Fig 4A). Figs 7A and 9C show that this 2*t*_*g*_ is present in the response of both the stapes and the BM when the cochlea is focally perturbed. However, when the cochlea is prescribed with random perturbations, the stapes does not show a strong pattern associated with any one group delay due to the multiple random reflections from the entire cochlea.

## Discussion

In this work we have shown that intracochlear perturbations generate codas in the temporal response of the BM. In models representing a healthy cochlea, the amplitude and duration of the coda depend on the profiles of random perturbations, consistent with experimental observations that the secondary response varies from cochlea to cochlea. The coda is present regardless of the excitation method, but varies from place to place. We also find that under a focal intracochlear perturbation, the stapes exhibits a coda similar to that in the BM.

In this section, we discuss the underlying causes for the diversity of the coda. First we discuss compression and traveling waves with a special focus on the manner in which wave reflection leads to the formation of the secondary response. We then analyze the relation between the spatial variation of the random profile and the presence or absence of codas. Finally, we consider extensions of this work for use as a non-invasive diagnostic tool for hearing problems.

### Intracochlear compression wave

During conventional acoustic excitation, a sound pressure wave is transmitted from the ear canal to the stapes, launching a forward traveling wave on the BM that peaks at different longitudinal locations according to the frequency content of the sound. A complement to the traveling wave is an intracochlear compression wave, which is generated due to the volumetric change at the stapes. It travels at the speed of sound in water, and does not contribute to the motion of the BM in our present model. Under internal laser heating, fluid thermal expansion induces the compression wave as well. This wave propagates both to the apex and to the base, away from the heat source. Due to the reflection at the boundaries, the compression wave travels back and forth multiple times in the cochlear channel before vanishing, causing periodic-like spikes of the stapes [20]. When the heat induced compression wave reaches the stapes for the first time, it initiates the response of the stapes, which in turn induces a forward traveling wave on the BM just as the conventional acoustic pathway. In this case, the group delay of the primary wave packets in Fig 8 is (*t*_*g*_ + 1.7*μ*s). Since 1.7*μs* ≪ *t*_*g*_, this explains the nearly identical group delays seen in Figs 4 and 8.

We investigated the influence of the boundary conditions at the basal end on the cochlear responses. When a *ρc* boundary condition, an impedance matching condition for a plane wave or an infinite tube, was imposed at both the stapes and round window along with a pair of symmetric, internally applied pressure sources above and below the BM, we found, as expected, that no reflected compression wave arose and a train of spikes at the stapes was absent (i.e., the compressional wave passed through the perfectly matched boundary). The BM remained undisturbed in this case.

### Retrograde traveling wave, temporal gaps between adjacent wave packets, and non-smooth patterns in the spectrum

**Fig 10:**
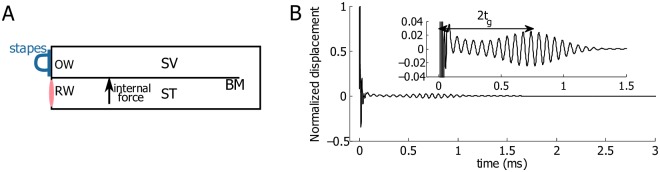
The propagation path and time of the intracochlear traveling wave. A: Schematics of internal impulse force excitation on the BM at a focal longitudinal position. This method is a mathematical idealization of a forced magnetic bead on the BM [22]. B: Normalized temporal displacement of BM at 2.5 mm under an internal force on the BM at 2.5 mm. The inset shows the zoomed-in response around 0.8 ms.

In our model, an internal focal force excitation on the BM (see Fig 10A) generates a large initial peak followed by a wave packet that arrives at time of 2*t*_*g*_ (0.82 ms) as shown in Fig 10B where the BM displacement at 2.5 mm to internal impulse force at the same location is plotted (a close-up of the delay response is shown in the inset). No wave packet arrives at a time *t*_*g*_ because the pure force does not cause a fast wave in our model [20]. The 2*t*_*g*_ delay results from propagation from the point of excitation to the base (denoted as a retrograde traveling wave) which has a delay of *t*_*g*_ plus the time it takes that wave to return to the point of force excitation (an additional *t*_*g*_ delay).

When the perturbation profile is random and distributed, our model predicts that coherent reflection gives rise to wave packets separated by gaps different from 2*t*_*g*_. For example, in Figs 4E and 5A, the gaps between the wave packets are greater than 2*t*_*g*_, while in Fig 5B the gaps are shorter and shows wax and wane. The varying or dynamic temporal gaps of echoes were observed in many experiments of BM measurements under acoustic clicks [1, 2, 23, 24] and electric impulses [25], as well as in the experimental results shown in Fig 6C. In some preparations with low sound pressure levels, the secondary responses beyond the primary wave packet have longer tails [1]. From our model predictions, long tails are predicted when the model activity or strength of random perturbations pushes the model close to the limit of stability. Taken together, the data and our model prediction suggest that in sensitive cochleae random perturbations may exist throughout the cochlear partition, which scatter and reflect waves at multiple places.

Reflection of the retrograde traveling wave was once thought to depend on the impedance at the stapes [14, 26]; certainly the OAE will depend on this boundary condition. We investigated the effect of altering the stapes boundary condition on the BM displacement coda. Once again, we applied a *ρc* boundary condition to stapes and round window and excited the cochlea using a normal acoustic click. Although a shift from the standard impedances of the round and oval window (fairly rigid and reactive, see Table 1) to the purely real *ρc* impedance is quite dramatic, this impedance does not diminish the amplitude of the secondary coda relative to the primary wave packet (see S1 Fig for the results from acoustic stimulation). Our theoretical prediction that the coda is not strongly influenced by the stapes boundary condition is consistent with the observation of Shera and Cooper [3] who modified the stapes boundary condition by placing a mass on the manubrium near the umbo. They too found that that this did not demonstrably affect the coda in their experiments. Hence, the coda appears to arise from interactions on the BM.

Fig 8 shows the model prediction from two slightly asymmetric heating sources. Although a resultant force is generated on the BM, the primary wave packet arrives at 2*t*_*g*_ rather than *t*_*g*_ because the resultant force is small and the compression wave dominates. If, however, the degree of asymmetry is increased so that the net force on the BM is increased, an additional wave packet at 2*t*_*g*_ will become more evident. In a healthy cochlea, a net force on the partition can potentially be generated from various physiological process, such as the distortion product that is hypothesized to arise from the interaction of two primary tones in a region near the best place of the higher tone [27]. In an experiment reported by He *et al.* [28], the BM only had a group delay *t*_*g*_ when it was stimulated by such forces. This could mean that in the experiment a dominant compression wave was intracochlearly generated in the process [19]. In our current work, we do not have a mechanism for intracochlearly generated compression wave (as opposed to the laser heating as an external source [19]); He *et al.* [28] suggested that such a mechanism exists, which we view as an open question.

The amplitude ripples and phase jumps in the spectrum are the counterpart of the coda in the temporal domain; this can be seen from Figs 4, 6 and 7 as well as from several physiological measurements [1–3, 23, 24]. Sharper ripples and peaks in the amplitude spectrum indicate more pronounced secondary coda, while removal of the secondary coda smoothens the BM spectrum (also see Shera and Cooper [3], for example). The half-cycle phase jumps in the spectrum is another feature of the pronounced secondary temporal coda; this phase-coda relation is consistent with reports from physiological measurement [3].

### Individual differences in the coda depend on the local wavenumber spectrum

We analyzed the temporal responses of the BM under two exemplary random profiles, and found that two seemingly similar roughness profiles (see Fig 3) gave rise to remarkably different codas. To determine the cause of this difference we analyzed the roughness through further processing. Coherent reflection is most effective when the wavelength of the random profile is half the wavelength of the traveling wave at a given location [13, 14]. The wavelength at each longitudinal location can be approximately identified by the predicted spatial BM response under pure tone acoustic simulation (at the characteristic frequency of the corresponding location). From our model, the wavelength at 2.5 mm is about 0.4 mm; the wavenumber corresponds to the half wavelength, i.e., *k* = 2*π*/(*λ*/2), is about 31.4 mm^−1^. The wavelength at 4.0 mm is about 0.45 mm; the wavenumber corresponds to the half wavelength is about 28 mm^−1^. In Fig 3B and 3D, where the spectrum were calculated from the entire cochlea, no significant difference can be found in the spectrum at 31.4 mm^−1^ and 28 mm^−1^ between R1 and R2. To investigate the localized property of the perturbation, we will restrict our wavenumber analysis within a small spatial window centered at location of interest; 2.25–2.75 mm for the 2.5 mm location and 3.75–4.25 mm for the 4.0 mm location.

**Fig 11:**
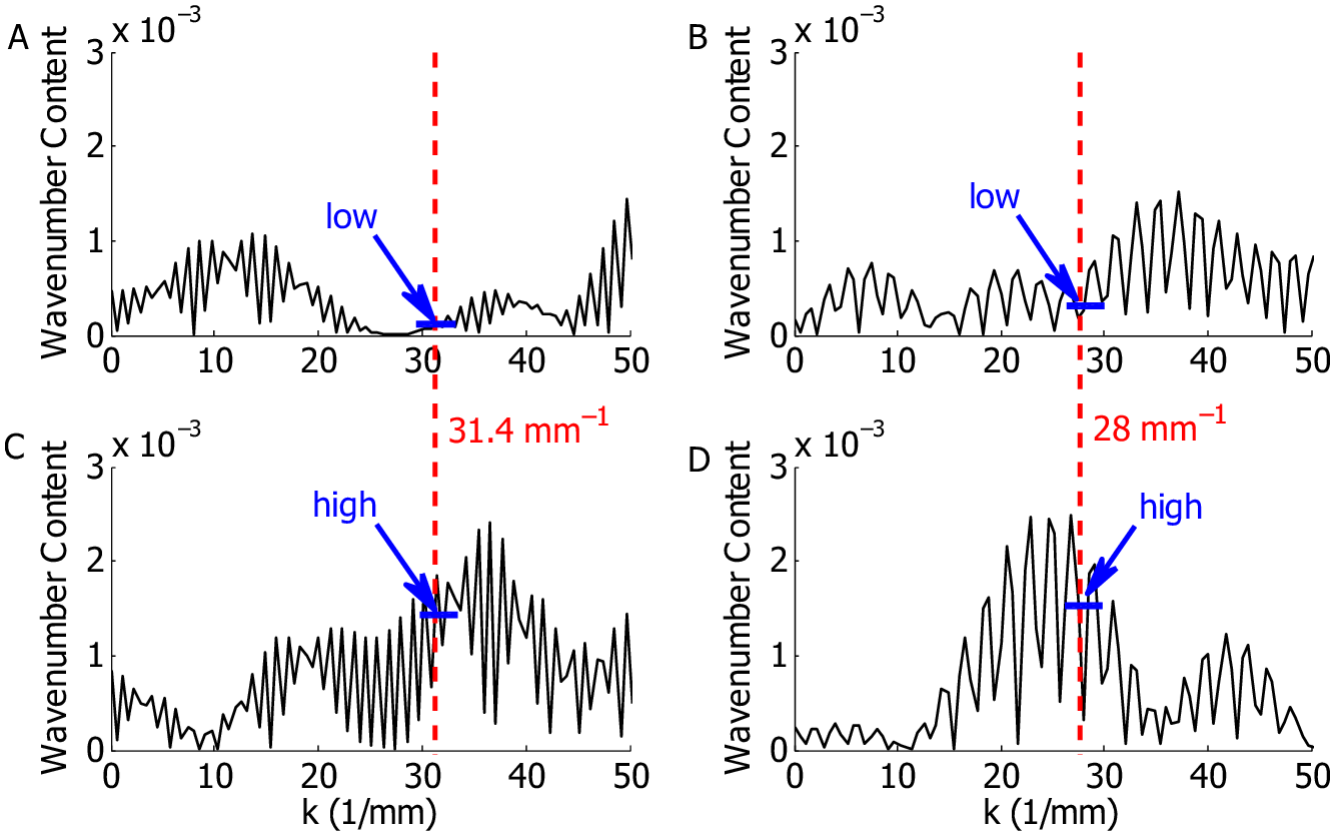
Localized spectra for R1 (A and B) and R2 (C and D). Fourier transformation is applied to filtered spatial profile and multiplied by 2*π*. A: The spatial profile in Fig 3A is computed for for *x* ∈ [2.25, 2.75] mm and filtered for the rest. B: The spatial profile in Fig 3A is computed for *x* ∈ [3.75, 4.25] mm and filtered for the rest. C: The spatial profile in Fig 3C is computed for for *x* ∈ [2.25, 2.75] mm and filtered for the rest. D: The spatial profile in Fig 3C is computed for *x* ∈ [3.75, 4.25] mm and filtered for the rest.

Fig 11 shows the filtered spectra of random profiles centered at these two locations and these spectra reveal the reason for the small coda under profile R1 and the larger coda under R2 (see Fig 4C and 4E). The spectrum of R2 centered at 2.5 mm has a high value (although not a peak) at 31.4 mm^−1^ (Fig 11C) but the spectrum of R1 is very low at 31.4 mm^−1^ (Fig 11A). Similarly, the spectrum of R2 centered at 4 mm has a high value at 28 mm^−1^ (Fig 11D), which is not seen in R1 (Fig 11B). Therefore, based on our model prediction the existence of extended wave packet depends on the spectral content of a random profile at best places, which can vary along the longitudinal domain. We also expect this space–wavenumber distribution to be idiosyncratic.

Furthermore, the extended wave packets disappear if the activity of the model is reduced (in our simulations we reduced activity by reducing the MET channel sensitivity). The individual difference and the vulnerability of the extended wave packets were observed in experiments as well. Kemp [4] measured the signal in the ear canal and showed different temporal patterns across individuals (the intracochlear wave reflections affect the motion of the stapes, and thus the emission in the ear canal). In the results reported by Recio *et al.* [1] and Rhode [2], various temporal vibrations of the BM were also observed beyond the primary wave packet. Some of these secondary responses displayed repeated echoes in a systematic way, similar to the response in Fig 5B, while others showed minimum echoes, as seen in Fig 4C. In another experiment by Parthasarathi *et al.* [6], an injection of current—thus increasing the cochlear gain—amplified the echo response.

Although our results, which replicate the qualitative aspects of the coda, suggest that random roughness underlies coda generation, however, the conclusion that this roughness is the exact mechanism that generates the coda must be made with caution; a rigorous proof of causation remains to be made experimentally to verify the correlation between the random profiles and the echo patterns. Our results and the interpretations are model-dependent; other very different models with random perturbations also have produced qualitatively similar results [3, 13, 14]. Experimentally the coda is a hallmark of a very sensitive cochlea. One cannot smooth out a biological cochlea, but added roughness might provide a way to test this hypothesis.

### Implication on non-invasive diagnoses

In this work, random perturbations are superimposed on the MET sensitivity to represent physiological irregularities developed in the cochlea. These perturbations can also be applied to other parameters, such as the stiffness of the BM, to indicate any possible morphological change on the cochlear partition. Our model predicts that regardless of the source of perturbation, when the cochlear activity is reduced, the amplitudes of the secondary responses decrease correspondingly. For a cochlea with fixed activity, when the strength of a perturbation is reduced, the amplitudes of the secondary responses decrease as well. In the model we use 12% for the local, singular perturbation and 2.0% for the random perturbations to maximize the secondary response while keep the model stable. For less active cochleae, such as unhealthy ones, the strength of perturbations can be increased without destabilizing the model. Hence, the secondary response is a combined effort of both the health of a cochlea and the strength of the perturbations.

Otoacoustic emissions (OAEs) has been used as a non-invasive assessment of the health of a cochlea for the dependence of OAE on the physiological activity of OHCs [10], but knowing the longitudinal location(s) of damaged or impaired OHCs is challenging. Our model prediction may overcome the difficulty: if the cochlea is perturbed at a focal longitudinal location, the response of the stapes under an acoustic click shows systematically delayed coda in the temporal domain and localized fluctuations in the frequency domain (see Fig 9C and 9D). This information can be used to identify the longitudinal location of the damage with the known frequency-location or the group delay-location mapping. When two or more focal perturbations exist in the cochlea, the temporal pattern of the stapes contains multiple overlapped wave packets; the spectrum of the stapes has fluctuations around the characteristic frequency of each focal perturbation. Although we are able to predict the response of the stapes with localized intracochlear damage, OAE, on the other hand, measures the emitted signal in the ear canal transmitted by the middle ear. Hence, non-invasive diagnoses with OAE requires incorporation of the knowledge of the middle ear transfer function (e.g., [15]).

In addition to identifying the location of local damage, the coda has implications on psychoacoustics. For example, repeated echoes on the BM indicates successive stimulations of the OHCs and thus the auditory nerves. The echoes have been described as a form of local, short term memory which may influence the ability of the auditory system to detect gaps in an acoustic stimulus such as speech [9] or may even set the just noticeable difference in frequency [16]. The strength and duration of the echoes vary along the cochlear axis, as shown in Figs 4 and 5, leading to different sound perceptions at different frequencies. In a healthy and stable cochlea the echoes, when exist, only last for a few milliseconds, but when the cochlea is unstable, the echoes have extended tails which may suggest a cochlear capable of spontaneous OAEs.

However, as our model predicts, in some cochleae the random, morphological variation on the cochlear partition does not lead to echoes if the wave is not scattered in a coherent manner. Hence, a lack of temporal coda does not imply the unhealthy of a cochlea. We expect further research on how the individuality of random perturbation in each cochlea can be applied to enable personalized diagnose and treatment of hearing problems. Overall, our model suggests that the coda on the BM is more of a fingerprint that is unique to individual cochlea rather than a universal feature.

## Supporting Information

S1 Table The rest parameters for the cochlear model. Here we list the parameters that are used in the cochlear model but have not been explicitly mentioned in the main text. *x* is in meters. BM: basilar membrane. TM: tectorial membrane. RL: reticular lamina. HB: hair bundle. OHC: outer hair cells. MET: Mechanoelectrical transducer. OW: Oval window. RW: Round window. SV: Scala Vestibuli. SM: Scala Media. ST: Scala Tympani.
(PDF)

S1 Fig The responses of the BM to acoustic stimulation using the typical and *ρc* stapes and round window boundary conditions are compared. Under the *ρc* boundary condition, the amplitude of the stapes is reduced by a factor of 6.4. In the figure the normalization ratio of the BM between the normal and the *ρc* response is 4.3:1. The relative amplitudes and timing of the wave packets (primary and coda) are nearly the same despite this change from a reactive original impedance to the resistive *ρc* impedance. Results are show for the BM displacement at 3.5 mm with random profile R2).
(EPS)
