# Supplementary material for: The Coda of the Transient Response in a Sensitive Cochlea: A Computational Modeling Study
Source: PLoS Comput Biol. 2016 Jul 5;12(7):e1005015. doi: 10.1371/journal.pcbi.1005015 (PMC4933343; doi:10.1371/journal.pcbi.1005015)
Supplement: S1 Table — Here we list the parameters that are used in the cochlear model but have not been explicitly mentioned in the main text. x is in meters. BM: basilar membrane. TM: tectorial membrane. RL: reticular lamina. HB: hair bundle. OHC: outer hair cells. MET: Mechanoelectrical transducer. OW: Oval window. RW: Round window. SV: Scala Vestibuli. SM: Scala Media. ST: Scala Tympani. (PDF) [file pcbi.1005015.s001.pdf]

Parameters	values
Fluid density	1×10^3 kg/m^3
Speed of sound in the fluid	1500 m/s
Helicotrema length	1 mm
BM width (b)	80 μm (base) to 180 μm (apex)
BM thickness (h)	7 μm (base) to 1.7 μm (apex)
HB length (L_{HB})	1 μm (base) to 6 μm (apex)
OHC length	25 μm (base) to 65 μm (apex)
TM radial length from attachment to middle-row OHC	87 μm (base) to 180 μm (apex)
Radial distance between OHCs	13 μm
TM longitudinal shear modulus	5 kPa
TM longitudinal shear damping	0.075 Pa s
Effective TM cross-sectional mass per unit length	$1.08 \times 10^{-6} e^{84.1x}$ kg/m
BM viscous damping per unit area	$0.1/b$ N s/m^3
TM radial bending damping per unit length	0.05 N s/m^2
TM radial shearing damping per unit length	0.03 N s/m^2
BM stiffness per unit area	$4.498 \times 10^9 (h/h_0)^4 (b_0/b)^4$ N/m^3
TM bending stiffness per unit length	$1.233 \times 10^4 e^{-672.7x}$ N/m^2
TM shear stiffness per unit length	$1.233 \times 10^4 e^{-672.7x}$ N/m^2
HB stiffness per unit length	$1.879 \times 10^4 e^{-706.4x}$ N/m^2
RL stiffness per unit length	$4.008 \times 10^4 e^{-706.4x}$ N/m^2
OHC stiffness per unit length	$4.008 \times 10^4 e^{-706.4x}$ N/m^2
Electromechanical coupling coefficient	-0.122 N/m/mV (base) to -0.152 N/m/mV (apex)
SV cable resistance per unit length	3 MΩ/m
SM cable resistance per unit length	5 MΩ/m
SV cable resistance per unit length	150 MΩ/m
Resistance between SV and ground	10 Ωm
Resistance between ST and ground	4 Ωm
Resistance between SV and SM	25 Ωm
OHC apical conductance per unit length	100 μS/m
OHC basolateral conductance per unit length	5100 μS/m (base) to 360 μS/m (apex)
OHC apical capacitance per unit length	50 nF/m
OHC basolateral capacitance per unit length	1800 nF/m (base) to 4200 nF/m (apex)
Potential difference between SM and OHC	$(150 - 1000x)$ mV
